# Supplementary material for: Effects of Artificial Light at Night on Fitness-Related Traits of Sea Urchin (Heliocidaris crassispina)
Source: Animals (Basel). 2023 Sep 27;13(19):3035. doi: 10.3390/ani13193035 (PMC10571867; doi:10.3390/ani13193035)
Supplement: Supplementary file 1 [file animals-13-03035-s001.zip › animals-2609746-supplementary.pdf]

Supplementary Information

Table S1: Water quality conditions during the experiment.

Time	ALAN groups	Parameter (Mean ± SD; n=3)		
		Temperature (°C)	Salinity (‰)	pH
1st week	0.1 Lux	24.96 ± 0.16	28.33 ± 0.91	6.75 ± 0.53
	300 Lux	25.02 ± 0.21	29.29 ± 1.11	6.79 ± 0.51
	600 Lux	25.05 ± 0.14	27.34 ± 0.80	6.86 ± 0.57
2nd week	0.1 Lux	25.07 ± 0.17	28.17 ± 1.01	6.83 ± 0.60
	300 Lux	24.98 ± 0.11	28.93 ± 0.71	6.82 ± 0.50
	600 Lux	24.99 ± 0.24	29.05 ± 0.90	6.83 ± 0.54
3rd week	0.1 Lux	25.03 ± 0.23	27.62 ± 0.91	6.79 ± 0.60
	300 Lux	25.07 ± 0.13	28.08 ± 1.16	6.78 ± 0.60
	600 Lux	24.98 ± 0.15	29.81 ± 0.88	6.84 ± 0.51
4th week	0.1 Lux	24.81 ± 0.17	27.32 ± 0.81	6.90 ± 0.52
	300 Lux	25.00 ± 0.17	29.67 ± 1.01	6.76 ± 0.56
	600 Lux	24.96 ± 0.12	28.01 ± 0.60	6.77 ± 0.57
5th week	0.1 Lux	25.07 ± 0.21	28.75 ± 1.71	6.84 ± 0.49
	300 Lux	25.11 ± 0.16	27.95 ± 1.11	6.90 ± 0.59
	600 Lux	25.03 ± 0.19	28.15 ± 0.90	6.83 ± 0.52
6th week	0.1 Lux	25.00 ± 0.13	27.95 ± 0.96	6.77 ± 0.58
	300 Lux	24.89 ± 0.14	28.67 ± 0.88	6.82 ± 0.51
	600 Lux	25.06 ± 0.15	28.01 ± 0.63	6.80 ± 0.60

***Procedure (Sp)*:** Cloning and analysis of full-length Pax6 gene

The total RNA was isolated from tubular feet (40–60 mg) of sea urchins *Heliocidaris crassispina* using Trizol Reagent (Invitrogen, Carlsbad, CA). The integrity and quantity of the extracted RNA were confirmed by agarose gel electrophoresis and spectrophotometry (Nano Drop 2000, Thermo Scientific, Delaware, USA). Reverse transcription was conducted with Prime Script@RT reagent Kit with gDNA Eraser (Takara, Kusatsu, Shiga, Japan) and the 3' and 5' ends of cDNAs were cloned using the RACE-PCR with the SMART RACE Kit (Clontech, Mountain View, USA). The universal amplified primers listed in *Table S2* were designed based on distinct conserved sequences from other species: *Strongylocentrotus purpuratus* (XM_030989294.1), *Octopus bimaculoides* (FJ876142.1), *Aedes aegypti* (XM_021838870.1) and *Ciona intestinalis* (NM_001032469.1). The PCR reaction was carried out with LA Taq DNA polymerase (Takara) in a total volume of 50 μL at 94 °C for 3 min, then 35 cycles of 94 °C for 1 min, 54 °C for 1 min, and 72 °C for 5 min, followed by a final extension of 72 °C for 10 min. Primers for end amplifications listed in *Table S2* were designed based on the partial fragment sequence of Pax6 and the RACEs were conducted according to the manufacturer's instructions (Clontech, USA). The PCR products were purified by using a San Prep Column DNA Gel Extraction Kit (Sangon, Shanghai, China) and loaded into the pMD 19-T cloning vector (Takara). At least three clones for each segment were sequenced from both directions (Invitrogen, Carlsbad, CA). These sequences were assembled into a single contig assembly using Sequencher v5.0 software (Gene Codes, Ann Arbor, MI, USA), and the assembly was then compared for identity against previously published Pax6 sequences in the National Center for Biotechnology Information (NCBI) BLAST program (http://blast.ncbi.nlm.nih.gov/). The Pax6 amino acid sequence was deduced using the ORF Finder (https://www.ncbi.nlm.nih.gov/ offender/) and aligned using ClustalW2 (http://www.ebi.ac.uk/Tools/clustalw2/) to available Pax6 sequences of other species obtained from the NCBI protein databases (https://www.ncbi.nlm.nih. gov/protein/). The protein domains were analyzed by the Simple Modular Architecture Research Tool (SMART) (http:// smart.embl-heidelberg.de/). The phylogenetic tree was constructed using the neighbor-joining (NJ) method in MEGA v.7 software. Confidence values for nodes were obtained by bootstrapping (1000 replicates).

Table S2. Nucleotide sequences of the primers for PCR

Primer names	Sequence (5'→3')	Target gene	Application
Pax6-uaF	CYAAYTCRAARTCCAGAVCAGAATA*		Cloning of partial fragment
Pax6-uaR	TCTTYCTCTRTVCNCTDCCTCTTTC*		cDNA
Pax6-3' outer	CCATCTATTTGAATCGCCTTAGAAC	Pax6	First-round 3'-RACE PCR
Pax6-3' inner	GAGAGATTTGTTGAAAGAGGAAGAG		Nested 3'-RACE PCR
Pax6-5' outer	TTGCCAAGTGTTCAGGTAA		First-round 5'-RACE PCR
Pax6-5' inner	CATGGTTACGATTCTCTTTACTCTG		Nested 5'-RACE PCR

Pax6: paired box 6 gene; RACE: rapid amplification of cDNA ends.

*N represents all four nucleotides; D: A, T or G; R: G or A; Y: C or T; H: A, T or C; V: G, A or C.

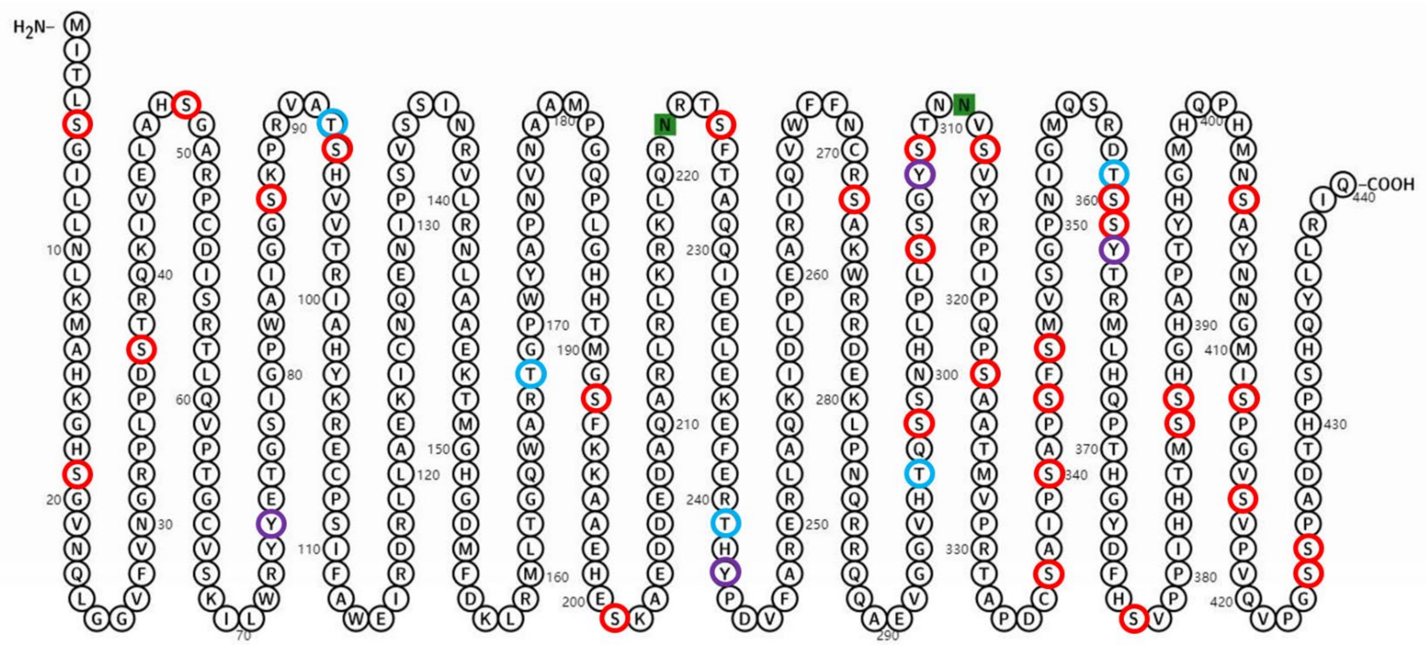

Figure S1. The deduced amino acid sequences of Pax 6 from *H. crassispina.* Two potential glycosylation sites (N222 and N312) in green and 38 potential phosphorylation sites, including 29 serine residues (S5, S19, S37, S48, S87, S94, S192, S201, S225, S272, S298, S305, S309, S314, S323, S336, S340, S343, S345, S360, S361, S377, S386, S387, S404, S412, S416, S424, S425) in red, 5 threonine residues (T93, T168, T241, T296, T359) in blue, and 4 tyrosine residues (Y74, Y243, Y308, Y362) in purple.

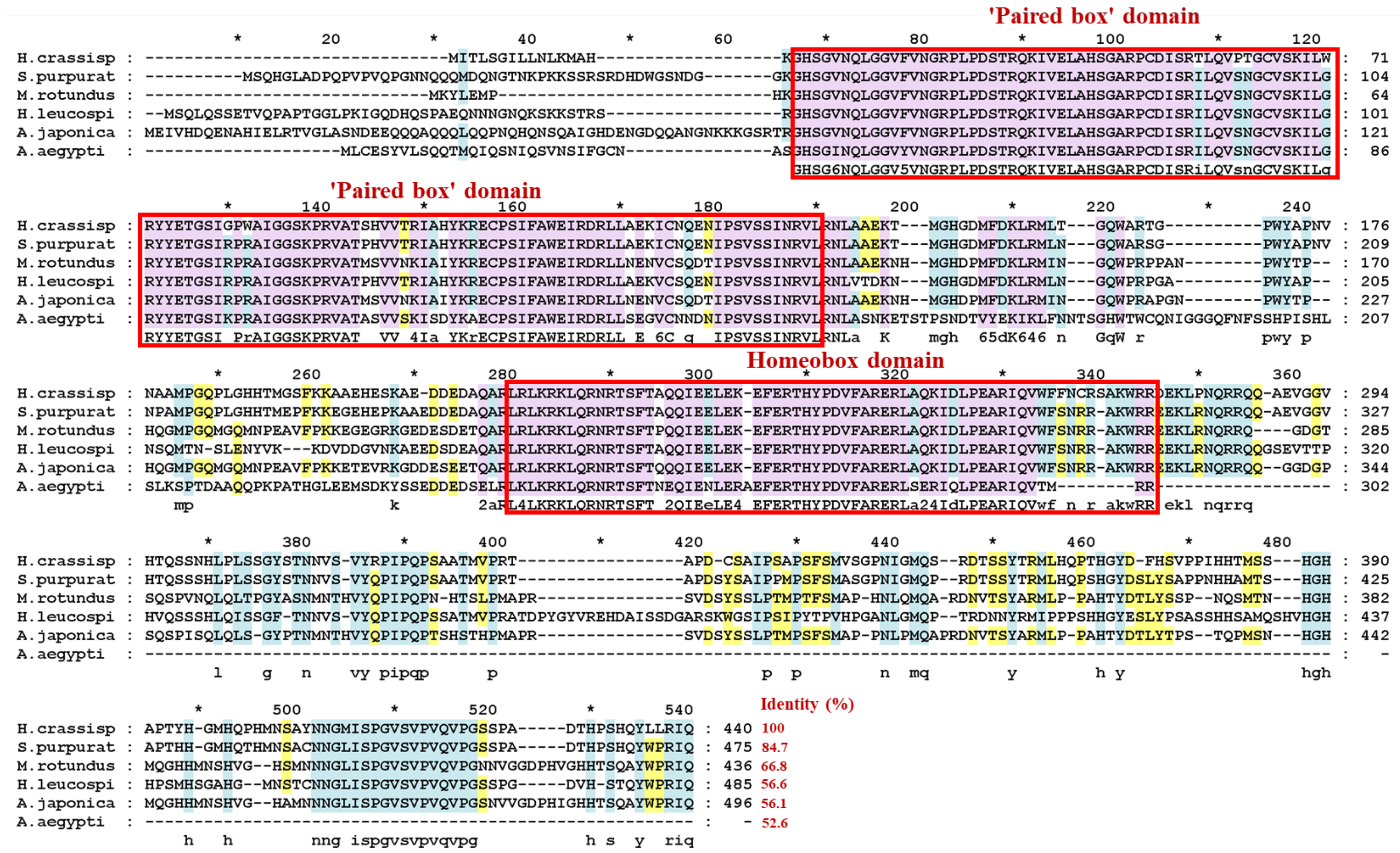

Figure S2. Alignment of deduced amino acid sequences of Pax6 from *H. crassispina*, *Strongylocentrotus purpuratus* (XP_030845151), *Metacrinus rotunda* (ADE59459.10), *Holothuria leucospilota* (KAJ8023790.1), *Anneissia japonica* (BBH63270.1) and *Aedes aegypti* (XP_021694562). The ‘Paired box’ domain and Homeobox domain are indicated using boxes. The sequence identities of the Pax6 protein between *H. crassispina* and other species are indicated at the end of this figure.

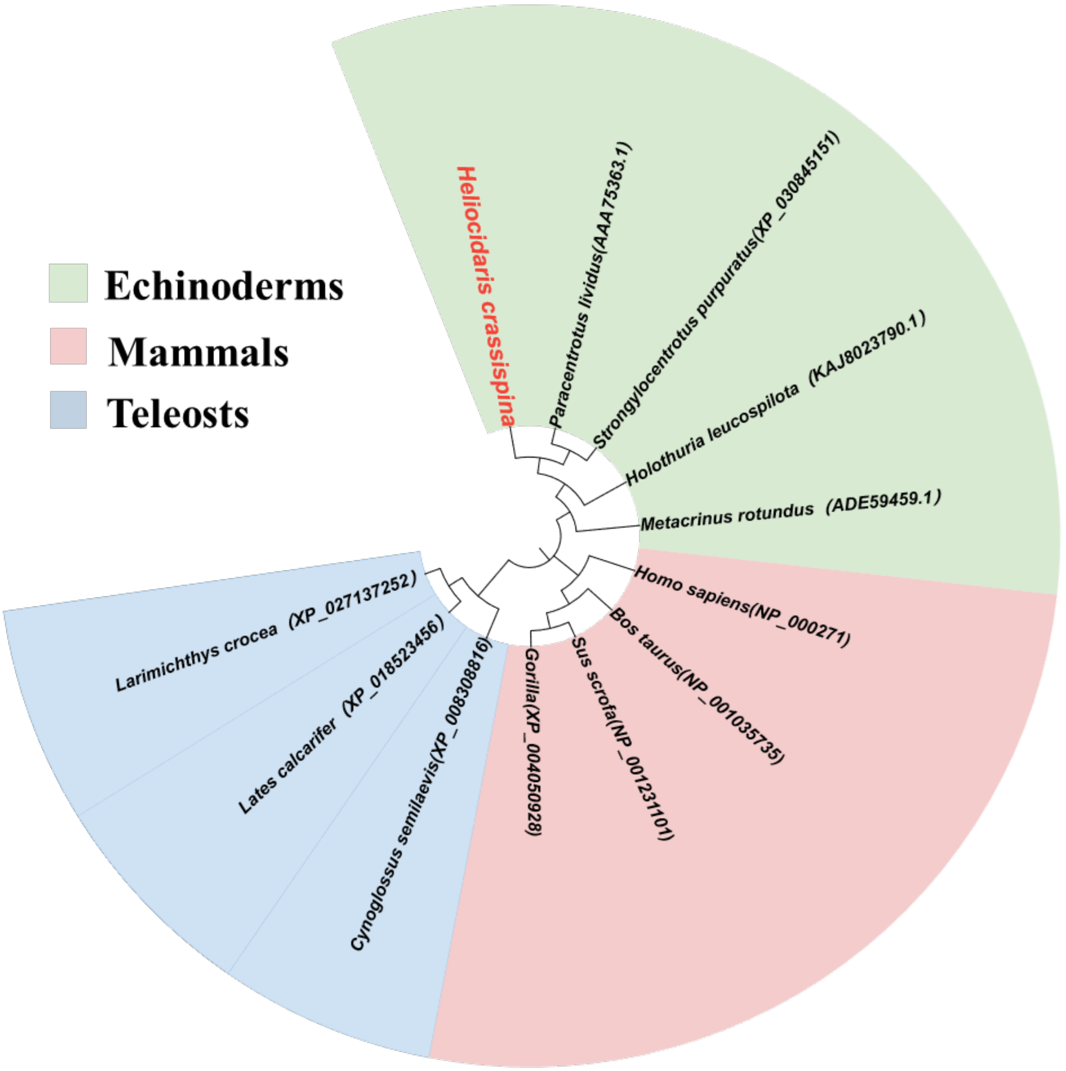

Figure S3. Phylogenetic tree illustrating the relationship of Pax6 from *H. crassispina* and select species. The Pax6 from *H. crassispina* is marked in red. All the Pax6 of echinoderms were clustered in one branch, while the Pax6 of teleosts and mammals were clustered in other branches. The Pax6 of *H. crassispina* was closely clustered to that of sea urchin species. The phylogenetic tree can reveal the evolutionary relationship among species.
